# Neural network modelling reveals changes in directional connectivity between cortical and hypothalamic regions with increased BMI

**DOI:** 10.1038/s41366-021-00918-y

**Published:** 2021-08-02

**Authors:** Katharina Voigt, Adeel Razi, Ian H. Harding, Zane B. Andrews, Antonio Verdejo-Garcia

**Affiliations:** 1 School of Psychological Sciences and Turner Institute for Brain and Mental Health, Monash University, Melbourne, VIC Australia; 2 The Wellcome Centre for Human Neuroimaging, University College London, London, UK; 3 Department of Electronic Engineering, NED University of Engineering and Technology, Karachi, Sindh Pakistan; 4 CIFAR Azrieli Global Scholars Program, CIFAR, Toronto, ON Canada; 5 Department of Neuroscience, Central Clinical School, Monash University, Melbourne, VIC Australia; 6 Monash Biomedical Imaging, Monash University, Melbourne, VIC Australia; 7 Biomedicine Discovery Institute and Department of Physiology, Monash University, Melbourne, VIC Australia

**Keywords:** Obesity, Risk factors

## Abstract

**Background/Objectives:**

Obesity has been ascribed to corticostriatal regions taking control over homeostatic areas. To test this assumption, we applied an effective connectivity approach to reveal the direction of information flow between brain regions and the valence of connections (excitatory versus inhibitory) as a function of increased BMI and homeostatic state.

**Subjects/Methods:**

Forty-one participants (21 overweight/obese) underwent two resting-state fMRI scans: after overnight fasting (hunger) and following a standardised meal (satiety). We used spectral dynamic causal modelling to unravel hunger and increased BMI-related changes in directed connectivity between cortical, insular, striatal and hypothalamic regions.

**Results:**

During hunger, as compared to satiety, we found increased excitation of the ventromedial prefrontal cortex over the ventral striatum and hypothalamus, suggesting enhanced top-down modulation compensating energy depletion. Increased BMI was associated with increased excitation of the anterior insula over the hypothalamus across the hunger and satiety conditions. The interaction of hunger and increased BMI yielded decreased intra-cortical excitation from the dorso-lateral to the ventromedial prefrontal cortex.

**Conclusions:**

Our findings suggest that excess weight and obesity is associated with persistent top-down excitation of the hypothalamus, regardless of homeostatic state, and hunger-related reductions of dorso-lateral to ventromedial prefrontal inputs. These findings are compatible with eating without hunger and reduced self-regulation views of obesity.

## Introduction

Obesity contributes to ~5% of deaths globally, reduces life expectancy by almost a decade [1] and accounts for a 2.8% loss to the global gross domestic product [2]. The prevalence of obesity has risen in parallel with increased access to energy-dense, highly attractive foods [3]. These food choices can overtake the neural mechanisms that regulate homeostatic-based eating and promote overconsumption [4]. Obesity is underpinned by abnormal interactions between homeostatic and non-homeostatic brain systems. The homeostatic system regulates eating based on energy needs and is associated with hypothalamus activity [5]. The hedonic system encodes non-homeostatic aspects of food, such as incentive salience and affective value and involves striatal, insular and medial prefrontal regions [6]. Three main neurobiological theories of obesity have been suggested: (i) the ‘hedonic eating’ model proposes that brain areas processing reward (e.g., striatum, medial prefrontal cortex) become dominant over those involved in energy homeostasis (e.g., hypothalamus) in response to food cues [5–7]; (ii) the ‘eating without hunger’ framework suggests that the neural regions processing salient external stimuli outweigh those involved in internal energy sensing [8]; and (iii) the ‘self-regulation’ view emphasises impaired top-down regulation of lower-level striatal and limbic regions [9, 10]. Understanding the interaction between the neural systems pinpointed by these theories is key to envisage new approaches to prevent and treat obesity.

Neuroimaging functional connectivity studies have the potential to improve our understanding of the interplay between homeostatic and non-homeostatic neural systems. Food choices activate brain regions involved in energy regulation (hypothalamus), interoception and inner experiences (insula, posterior cingulate cortex), reward processing (medial prefrontal cortex, ventral striatum) and cognitive control (dorso-lateral prefrontal cortex, anterior cingulate cortex) [11, 12]. Homeostatic states (hunger versus satiety) and body mass index (BMI) have been associated with the functional connectivity between the hypothalamus, striatum, insula and prefrontal cortex regions [5, 13–16]. Specifically, hunger, as compared to satiety, has been associated with increased resting-state connectivity between the insula, the posterior cingulate cortex and the ventromedial prefrontal cortex [17, 18]. Furthermore, BMI has been associated with increased connectivity between the hypothalamus and the striatum, the insula and the ventromedial prefrontal cortex at rest [19–22], and reduced connectivity between the prefrontal cortex and both the insula and the striatum during food valuation and choice tasks [11, 23]. These findings variously support different models of obesity. Increased activation of the ventral striatum and ventromedial prefrontal cortex aligns with higher reward sensitivity for food as proposed by the ‘hedonic eating’ model [7, 24]. Alternatively, lower connectivity between the dorso-lateral prefrontal cortex or anterior cingulate cortex and striatum or ventral medial prefrontal cortex aligns with limitations in cognitive control [12] as proposed by the ‘self-regulation’ model [9, 10]. And finally, disrupted connectivity involving the hypothalamus or insula/posterior cingulate cortex [17, 21, 25, 26] agrees with an ‘eating without hunger’ framework [8], in which internal homeostatic signals are disregarded. However, a critical limitation of all these studies is that they cannot speak to the direction of information flow between the brain regions (e.g., whether they reflect bottom-up or top-down communication) or the valence of the connections (whether they are excitatory or inhibitory). Such information is needed to reveal if/how, as suggested by neurobiological theories, non-homeostatic brain systems take over homeostatic areas.

Here, we applied a novel hypothesis-testing neural network modelling framework to unravel the direction and valence of connections between hypothalamic, striatal, insula and cortical regions as a function of BMI and energy homeostasis. We hypothesised that BMI would associate with increased influence of striatal and insula regions on the hypothalamus (‘hedonic eating’ framework) and decreased influence of cortical regions on lower-level areas (i.e., striatal and insula regions) (‘decreased self-regulation’ framework). Hunger would be associated with an adaptive increased impact on connectivity in interoceptive regions (insula/pCC), whereas the interaction with BMI would show a decreased impact on connectivity in interoceptive regions (‘eating without hunger’ framework).

## Methods

### Participants

Forty-one human participants (21 females, mean age = 24.37, SD = 5.53) were recruited from the general community. Participants’ BMI ranged from 18 to 38 kg/m^2^: 20 had healthy weight (18–24.9 kg/m^2^), 21 were overweight (BMI = 25–30 kg/m^2^) or obese (BMI > 30 kg/m^2^) (Table 1 for further descriptive data). An initial screening interview assured that these participants (1) had no history of hypertension or diabetes, (2) had no neurological and psychiatric illness or (3) were on psychoactive medication affecting cognitive functioning or cerebral blood flow. The number of participants was chosen based on a sample size estimation study revealing that 20 participants provided for reliable DCM predictions [27]. In agreement, recent research showed robust model predictions using similar sample sizes when applying spectral dynamic causal modelling (spDCM) to rsfMRI data [28–30]. All participants were naive to the purpose of the study, gave written consent before participating and were reimbursed with $100 gift card vouchers. The Monash University Human Research Ethics Committee approved the study (2019-5979-30222) following the Declaration of Helsinki.

**Table 1:** Participants’ demographics per BMI group.

	Healthy weight	Overweight	Obese
Age, mean (SD)	23.9 (5.61)	24.57 (1.17)	24.93 (6.39)
Gender (female/male)	10/10	3/7	8/6
BMI (kg/m^2^)	21.94 (1.94)	27.41 (1.17)	33.94 (3.08)

*SD* standard deviation, *BMI* body mass index.

### Experimental procedure

Participants completed two fMRI sessions, one after an overnight fast (hunger condition) and one after a standard breakfast (satiety condition). In both conditions, participants were instructed to have a standard meal (700–1000 kj) between 7.30 pm and 8.30 pm on the night prior to their scan, and refrain from eating or drinking (except for water) until their morning scan. For the satiety condition, participants received a standard breakfast (293 kcal) 1 h prior to their scan. Subjective self-reports of hunger (1 = not hungry at all; 7 = very hungry) revealed a significant difference in the perception of hunger between the hunger (M = 4.63; SD = 1.46) and satiety (M = 3.12; SD = 1.58) conditions (*t*(40) = 4.72, *p* < 0.001). There was no significant difference in subjective feelings of fullness across the weight groups when they received a meal (*t*(40) = –1.0, *p* = 0.32). We therefore did not include subjective feelings of fullness as a parametric regressors into our main spDCM analyses. Both fMRI sessions were scheduled in the morning between 9 am and 10 am and on average there were 5.82 days (SD = 3.73 days) between the two scanning sessions. Scheduling the participant’s first scan as either part of the hunger or satiety condition was counterbalanced across participants.

Each fMRI session was divided into an initial task-free fMRI (i.e., resting-state fMRI) sub-session and a subsequent task-based fMRI sub-session. A large body of literature suggests that functional connectivity at rest is altered by previous task experiences (e.g., [31, 32]). As such, task-based fMRI scans were run after the resting-state fMRI scans in order to avoid any artificial inflation of signal-to-noise ratio at rest. The full details of this session and results are outlined in further detail in a previous publication by our group [11], but all relevant sections are repeated here.

#### Resting-state fMRI sub-session

Resting-state fMRI data were acquired using a 3-Tesla Siemens Skyra MRI scanner equipped with a 32-channel head coil at the Monash Biomedical Imaging Research Centre (Melbourne, Victoria, Australia). During each 8-min scan, 189 gradient-echo planar images comprising of 44 interleaved, continuous axial slices were collected (repetition time = 2500 ms; echo time = 30 ms; flip angle = 90°; 3 mm isotropic voxels; field of view = 192 mm). A whole-brain T1-weighted magnetisation-prepared rapid gradient-echo structural image was also acquired for each session and each participant (192 sagittal slices; 1 mm isotropic voxels; repletion time = 2300 ms; field of view = 256 mm). Participants were instructed to rest while closing their eyes.

#### Task-based fMRI sub-session

Following the resting-state fMRI scan of each session, participants completed a food choice task in conjunction with functional MRI which results have been previously reported [11]. ROI selection for subsequent spDCM analyses was based on the results revealed during the task-based fMRI session (see details below and Table 2). The food choice task was designed to interrogate the neural basis of food choices using actual physiological stimuli. The task presented pseudorandomised images of pairs of healthy (low sugar and fat) and/or unhealthy (high sugar and fat) beverage combinations in the form healthy–healthy, unhealthy–unhealthy and unhealthy–healthy options. The beverage pair combinations were consistent and presented to all participants in the same order. In each trial, participants were asked to select an option based on their usual preferences. Each image pair was displayed for a 3-s viewing-only period followed by a 1.5-s period where participants made their selection using a two-button response box. The chosen beverage was delivered during the 5 s following selection. The task comprised five runs, each containing 30 choice events (10 from each condition) and lasting 6 min 32 s.

**Table 2:** Locations of regions of interests (spDCM nodes).

Region	Hemisphere	MNI coordinates			Source
		*x*	*y*	*z*	
pCC	R	2	–42	30	Harding et al. (2018)
dlPFC	L	–46	42	16	Harding et al. (2018)
aI	L	–38	12	–6	Harding et al. (2018)
dACC	L	–4	32	30	Harding et al. (2018)
Hypo	ML	0	–3	–12	Neurosynth
vStr	L	–4	8	–3	Batra et al. (2013)
vmPFC	R	12	58	22	Harding et al. (2018)

*pCC* posterior cingulate cortex, *dlPFC* dorso-lateral prefrontal cortex, *aI* anterior insula, *dACC* dorso-anterior cingulate cortex, *Hypo* Hypothalamus, *vStr* ventral striatum, *vmPFC* ventromedial prefrontal cortex, *MNI* Montreal Neurological Institute, *R* right, *L* left, *ML* midline.

A computer-controlled MRI-compatible food delivery system (gustometer), consisting of six syringe pumps each connected to a different beverage reservoir, was used to deliver the chosen beverages to participants during simultaneous fMRI recording. Upon beverage selection, 3 ml of the beverage was delivered via plastic tubing to a mouthpiece mounted on the head coil of the scanner.

The beverages were designed by a professional nutritionist and prepared according to a standard operating procedure. ‘Unhealthy’ beverages included chocolate, strawberry and caramel milkshakes. ‘Healthy’ beverages included fruit-blended orange, cranberry/raspberry and veggie juices (including fibre). The unhealthy drinks had significantly more sugar and fat than the healthy drinks: chocolate milkshake (8.93 g of fat and 18.99 g of sugar per 100 ml), strawberry milkshake (8.93 g of fat and 18.93 g of sugar per 100 ml), caramel milkshake (8.93 g of fat and 18.93 g of sugar per 100 ml) versus veggie juice (o1 g of fat and 7.7 g of sugar per 100 ml), cranberry/raspberry juice (o1 g of fat and 9.6 g of sugar per 100 ml) and orange juice (o1 g of fat and 8.4 g of sugar per 100 ml).

### Resting-state fMRI data analyses

#### Preprocessing

Functional images were pre-processed for each session separately using SPM12 (revision 12.2, www.fil.ion.ucl.ac.uk). The preprocessing steps consisted of slice time correction, realignment, spatial segmentation and normalisation to the standard EPI template of the Montreal Neurological Institute (MNI) and spatial smoothing using a Gaussian kernel of 8-mm FWHM. No (band-pass) filtering was used except a low-pass filter (of 1/128) that filters the ultra-low frequency scanner drifts [33]. None of the participants exceeded excessive head motion of larger than 3 mm in translation or 3mradians rotation.

#### ROI selection and time series extraction

**Fig. 1:**
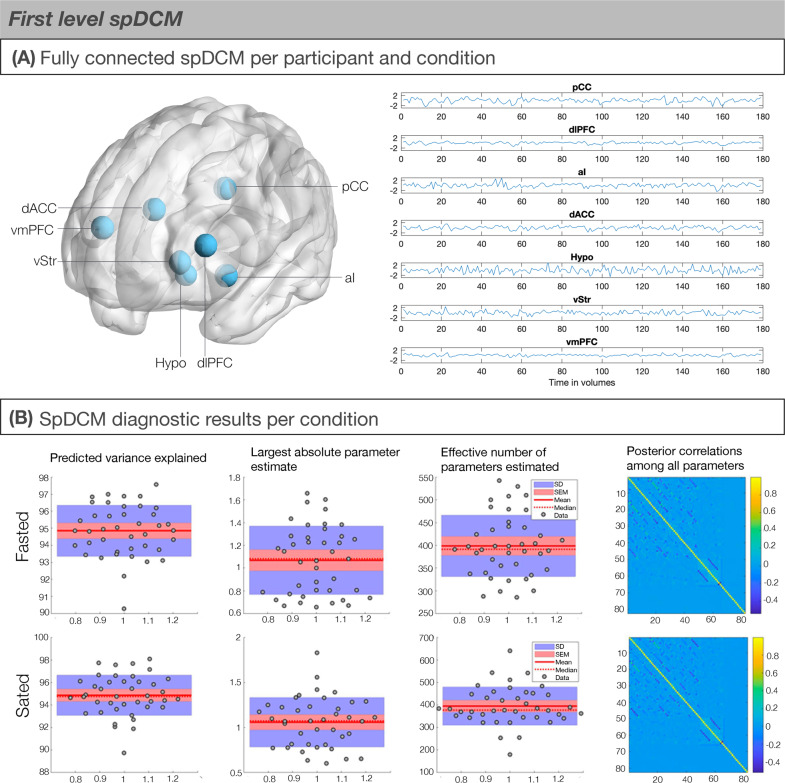
First-level spDCM modelling in the selected regions of interest. **A** Brain areas involved in regulated food choice. ROI selection of food choice network was based on task-based fMRI results within the same sample (Harding et al., 2018). (Left) Schematic showing the seven regions of interests (ROIs) used to estimate spDCMs with the fully connected architecture (i.e., 7^2^ = 49 parameter model; Friston et al., 2014). (Right) The time series of the ROIs for an exemplar subject. **B** First-level DCM model convergence statistics indicating good model convergence. (First column) Predicted variance explained for each individual was high mostly above 75%. (Second column) The largest absolute parameter estimate did not fall below the typical connection strength of 1/8 Hz. (Third column) The effective number of parameters are reported in terms of divergence between the posterior and prior densities over parameters. (Fourth or last column) Posterior correlations among all parameters were low, indicating identifiable parameters.

Seven ROIs that are featured in the reviewed neurobiological theories of obesity were identified as key nodes for effective connectivity analyses. The identified neural circuit comprised of the posterior cingulate cortex, dorso-lateral prefrontal cortex, anterior insula, dorso-anterior cingulate cortex, hypothalamus, ventral striatum and ventromedial prefrontal cortex (Fig. 1A). The MNI coordinates for the posterior cingulate cortex, dorso-lateral prefrontal cortex, anterior insula and dorso-anterior cingulate cortex were based on activity associated with food choices during the task-based fMRI session ([11], their Tables 1 and 2). The coordinates for the hypothalamus and ventral striatum were derived from quantitative meta-analyses, as they are part of the food choice network, but were not reported as significant clusters in this sample [11]. The coordinates for the ventral striatum were derived from a coordinate-based meta-analysis of BOLD fMRI experiments examining neural correlates of subjective value [34] and the hypothalamus’ coordinates were derived from the Neurosynth (www.neurosynth.com), as no quantitative meta-analysis on (food) decision making and the hypothalamus exists. We did not perform any diagnostic checks in order to check for potential artefacts in the hypothalamus ROI.

To extract BOLD fMRI time series corresponding to the aforementioned ROIs, the pre-processed data were used to establish the residuals of a General Linear Model (GLM). Six head motion parameters and WM/CSF signals were added to the GLM as nuisance regressors. Finally, we selected the MNI coordinates as the centre of a 6-mm sphere to compute the subject-specific principal eigenvariate and correct for confounds.

### Neural network modelling: spectral dynamic causal modelling (spDCM)

The spDCM analyses were performed using the functions of DCM12 (revision 7196) implemented in SPM12. In order to address our main hypotheses, we focused on spDCM analyses that assessed (1) changes in effective connectivity of hunger versus satiety condition independent of BMI (main effect of hunger), (2) changes in effective connectivity modulated by BMI (main effect of BMI) and (3) changes in hunger-related effective connectivity modulated by BMI (BMI-by-hunger interaction). Hunger condition was a grouping variable, whereas BMI was a continuous variable. BMI was treated as continuous, as opposed to a grouping variable, as our sample contained overweight (BMI = 25–30 kg/m^2^) and obese (BMI > 30 kg/m^2^) individuals.

#### First-level spDCM analysis

On the first level, a fully connected model was created for each participant and each session (i.e., 7^2^ = 49 connectivity parameters, including seven inhibitory self-connections). Next, we inverted (i.e., estimated) the DCMs using spectral DCM, which fits the complex cross-spectral density using a parameterised power-law model of endogenous neural fluctuations [33]. This analysis provides measures of causal interactions between regions, as well as the amplitude and exponent of endogenous neural fluctuations within each region [33]. Model inversion was based on standard variational Laplace procedures [35]. This Bayesian inference method uses Free Energy as a proxy for (log) model evidence, while optimising the posterior density under Laplace approximation of model parameters.

#### Second-level spDCM analysis

To characterise how group differences in neural circuitry were modulated by BMI and Hunger condition, hierarchical models over the parameters were specified within a hierarchical Parametric Empirical (PEB) framework for DCM [36]. The three models we used were based on our hypotheses as follows: first, we were interested in the group difference between fasted versus sated conditions and in this PEB analysis, we used BMI, age and gender as regressors of no interest. Second, we were interested associating effective connectivity with BMI and in this PEB analysis, we used group factor (fasted versus sated), age and gender as regressors of interest. Lastly, we were interested in interaction between group factor (fasted versus sated) and BMI and in this PEB analysis, we used BMI, group factor (fasted versus sated), age and gender as regressors of no interest.

Note, that for each of the presented models, all behavioural regressors were mean-centred so that the intercept of each model was interpretable as the mean connectivity. Hunger condition was modelled as the main regressor of interest as a vector consisting of 1 (fasted) and –1 (stated). The interaction term between BMI and Hunger Condition was created by first centreing the continuous variable BMI before creating the element-by-element product of the newly centred BMI variable with the categorical variable Hunger condition [37]. Hunger condition was a grouping variable, whereas BMI was a continuous variable. BMI was treated as continuous, as opposed to a grouping variable, as our sample contained overweight (BMI = 25–30 kg/m^2^) and obese (BMI > 30 kg/m^2^) individuals.

Bayesian model reduction was used to test all reduced models within each parent PEB model (assuming that a different combination of connections could exist [36] and ‘pruning’ redundant model parameters); parameters of the best 256 pruned models (in the last Occam’s window) were averaged and weighted by their evidence (i.e., Bayesian Model Averaging) to generate final estimates of connection parameters. To identify important effects (i.e., changes in directed connectivity), we compared models (using log Bayesian model evidence to ensure the optimal balance between model complexity and accuracy) with and without each effect and calculated the posterior probability for each model, as a softmax function of the log Bayes factor. We treat effects (i.e., connection strengths and their changes) with posterior probability >0.99 as significant for reporting purposes.

Finally, in order to determine the predictive validity (e.g., whether BMI can be predicted from the final, reduced spDCM’s individual connections), leave-one-out cross-validation was performed within the PEB framework [38]. This procedure fits the PEB model to all but one participant and predicts the covariate of interest (e.g., BMI) for the left-out participant. This is repeated with each participant to assess the averaged prediction accuracy for each model. The code is available on reasonable request by contacting the corresponding author. The experiment as it is has been conducted for the first time in our laboratory and no replicates exist yet.

## Results

At the subject level, we used the time series from the ROIs to define and estimate a fully connected DCM for each participant and condition using Variational Laplace [35] (Fig. 1A). Post-hoc diagnostic statistics ensured that first-level model inversion had converged (Fig. 1B). The average variance explained across subject-level DCM inversion was very high (Hunger: M = 94.85, SD = 1.50, range = 90.27–97.58; Satiety: M = 94.85, SD = 1.78, range = 89.73–98.06), indicating very good model fitting (Fig. 1B, first column). The largest absolute parameter estimate did not fall below the typical connection strength of 1/8 Hz (Hunger: Satiety: M = 1.07, SD = 0.30; Satiety: M = 1.06, SD = 0.27) (Fig. 1B, second column). The effective number of parameters is reported in terms of divergence between the posterior and prior densities over the parameters (Hunger: M = 398.68, SD = 67.52; Satiety: M = 394.30; SD = 84.67) (Fig. 1B, third column). Finally, the posterior correlations among all parameters were low, indicating identifiable parameters (Fig. 1B, final column).

### Homeostatic state (hunger versus satiety)

**Fig. 2:**
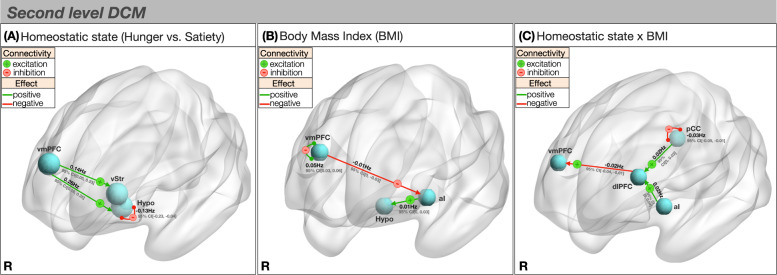
Second-level spDCM results. **A** Effective connectivity of hunger. Hunger was associated with increased excitatory connectivity from the ventromedial prefrontal cortex to the ventral striatum and hypothalamus and decreased hypothalamic self-inhibition. **B** Effective connectivity of BMI. BMI was associated with decreased inhibition from the ventromedial prefrontal cortex to the anterior insula, increased excitation from the anterior insula to the hypothalamus and increased self-inhibition of the ventromedial prefrontal cortex. **C** Effective connectivity of BMI × Hunger State. During hunger, compared to satiety, higher BMI was associated with decreased dorso-lateral prefrontal cortex to ventromedial prefrontal cortex excitation, increased excitation from the posterior cingulate cortex and anterior insula to the dorso-lateral prefrontal cortex and decreased posterior cingulate cortex self-inhibition. + or – signs code the parity of connectivity: – inhibitory, + excitatory. pCC posterior cingulate cortex, dlPFC dorso-lateral prefrontal cortex, aI anterior insula, Hypo Hypothalamus, vStr ventral striatum, vmPFC ventromedial prefrontal cortex, R right.

To explore how homeostatic state is associated with connectivity changes, we examined causal network dynamics during hunger and satiety while controlling for BMI. Starting from a fully connected model (Fig. 1), Bayesian optimisation procedures revealed a sparse model structure with a posterior probability of >0.99 at the group level (Fig. 2A and Table 3). Compared to satiety, hunger was associated with an increased excitatory influence of the ventromedial prefrontal cortex over the ventral striatum (0.14 Hz, 95% CI [0.05, 0.23]) and hypothalamus (0.26 Hz, 95% CI [0.16, 0.35]). We further found less self-inhibition (i.e., disinhibition) of the hypothalamus when individuals were hungry as opposed to sated (–0.13 Hz, 95% CI [–0.23, –0.04]). In general, self-inhibition models the recurrent inhibitory activity in the region [39]. Since by definition the self-connections are inhibitory, they suppress activity in the region so as to avoid any run-away excitation in the model. The self-inhibition of the hypothalamus might reflect a suppression of pathways that would normally cause satiety, such as the melanocortin pathway that is known to reduce food intake [40]. Leave-one-out cross-validation revealed that these effects from individual connections are large enough to predict left-out individuals’ hunger state above chance level (*r*(df = 80) = 0.32, *p* < 0.05). Cross-validation of this sort provides out of sample estimates of predictability (i.e., the predictive validity of the connectivity strength from a new participant’s hunger state).

**Table 3:** Summary of spDCM findings.

*Homeostatic state (hunger vs. satiety)*				
Parity	Connection	Hunger vs. satiety effect	Effect size in Hz [95% CI]	Posterior probability
Excitation	vmPFC → Hypo	+	0.26 [0.16, 0.35]	1
	vmPFC → vStr	+	0.14 [0.05, 0.23]	0.99
Inhibition	Hypo → Hypo	–	–0.13 [–0.23, –0.04]	0.99
*Body mass index (BMI)*				
Parity		BMI relationship	Effect size in Hz [95% CI]	Posterior probability
Excitation	aI → Hypo	+	0.01 [0, 0.03]	1
Inhibition	vmPFC → aI	–	–0.01 [0, –0.03]	1
	vmPFC → vmPFC	–	–0.05 [–0.03, –0.06]	1
	dACC → dACC	–	–0.02 [0, –0.03]	0.99
	dlPFC → dlPFC	–	–0.02 [–0.04, 0]	0.99
*Homeostatic state × BMI*				
Parity	Connection	Interaction effect	Effect size in Hz [95% CI]	Posterior probability
Excitation	aI → dlPFC	+	0.02 [0, 0.04]	0.99
	pCC → vmPFC	+	0.02 [0, 0.03]	0.94
Inhibition	dlPFC → vmPFC	–	–0.02 [–0.04, –0.01]	0.99
Inhibition	pCC→ pCC	–	–0.03 [–0.05, –0.01]	1

*pCC* posterior cingulate cortex, *dlPFC* dorso-lateral prefrontal cortex, *aI* anterior insula, *dACC* dorso-anterior cingulate cortex, *Hypo* Hypothalamus, *vStr* ventral striatum, *vmPFC* ventromedial prefrontal cortex, *MNI* Montreal Neurological Institute.

### Body mass index (BMI)

Next, we explored how BMI is associated with connectivity changes, whilst controlling for homeostatic state. Elevated BMI was associated with an increased excitatory influence of the anterior insula on the hypothalamus (0.01 Hz 95% CI [0, 0.03]) and a reduced inhibitory influence of the ventromedial prefrontal cortex on the anterior insula (–0.01 Hz, 95% CI [0, –0.03]) (Fig. 2B and Table 3). In addition, individuals with greater BMI had increased self-inhibition of the ventromedial prefrontal cortex (0.05 Hz, 95% CI [0.03, 0.06]) and dorso-anterior cingulate cortex (0.02 Hz, 95% CI [0, 0.03]), and decreased self-inhibition of the dorso-lateral prefrontal cortex (–0.02 Hz, 95% CI [–0.04, 0]) and posterior cingulate cortex (–0.02 Hz, 95% CI [–0.03, 0]). Self-inhibition of ventromedial prefrontal cortex, dorso-anterior cingulate cortex and dorso-lateral prefrontal cortex associated with increased BMI is likely to reflect different changes in interneuron activity. These differences are to be expected as there are different influences of GABAergic interneurons under these conditions. Leave-one-out cross-validation revealed that these effects sizes from individual connections are large enough to predict group effects with an out of sample estimate (*r*(80) = 0.22).

### Interaction of BMI and homeostatic state

In the final analysis, we investigated how hunger-related connectivity changes may be associated with differences in BMI. During hunger relative to satiety, higher BMI was associated with a lesser excitatory influence of the dorso-lateral prefrontal cortex over the ventromedial prefrontal cortex (–0.02 Hz, 95% CI [–0.04, –0.01]) and a greater excitatory influence of the anterior insula over the dorso-lateral prefrontal cortex (0.02 Hz, 95% CI [0, 0.04]) (Fig. 2C and Table 3). In addition, we found decreased self-inhibition of the posterior cingulate cortex (–0.03 Hz, 95% CI [–0.05, –0.01]), potentially reflecting different influences of GABAergic interneurons. An increased excitatory influence of the posterior cingulate cortex on the dorso-lateral prefrontal cortex was also evident below the set posterior probability threshold of >0.99 (0.02 Hz, 95% CI [0, 0.03], posterior probability = 0.94). The out of sample correlation between the model’s prediction and observed data was significant as revealed by leave-one-out cross-validation (*r*(80) = 0.19).

## Discussion

**Fig. 3:**
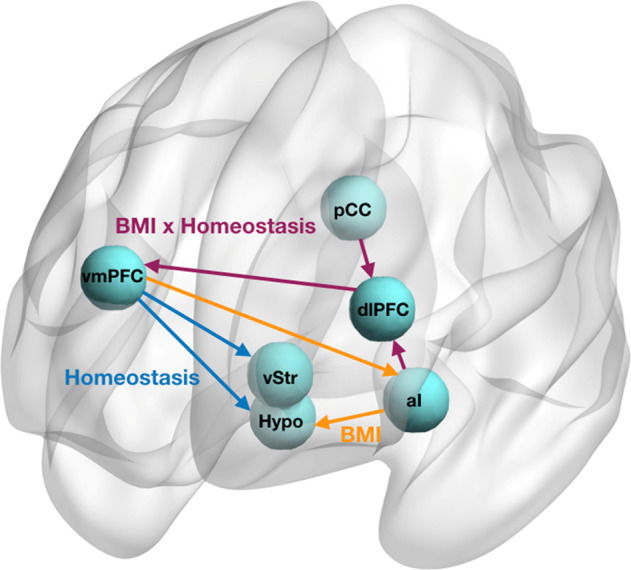
Overview of spDCM results. Schematic diagram summarising the resting-state neuronal network configurations associated with homeostasis, BMI and homeostasis × BMI interaction effects.

This study reveals novel obesity-related changes in directional interactions between corticostriatal and homeostatic regions (summarised in Fig. 3). We specifically examined brain regions featured in neurobiological theories of obesity, including the hedonic eating, eating without hunger and self-regulation views. We found that higher BMI was associated with a greater excitatory influence of the anterior insula on the hypothalamus, regardless of homeostatic state (i.e., during both hunger and satiety). This finding is consistent with reduced sensitivity to changes in energy homeostasis and an eating without hunger view [8–10]. Furthermore, participants with higher BMI showed weaker excitatory influence of the dorso-lateral prefrontal cortex on the ventromedial prefrontal cortex during the hunger state. The interaction between these two regions has been previously associated with dietary self-regulation [12]. In addition, we showed that, regardless of adiposity, during hunger as compared to satiety the ventromedial prefrontal cortex increased its excitatory influence over the ventral striatum and the hypothalamus. This may represent a general adaptive mechanism of top-down signalling during energy deprivation [13].

Together with the hypothalamus, the anterior insula has been proposed to form a homeostatic/interoceptive network. This network prompts eating during energy deprivation and ends feeding upon satiation in humans [17] and plays a central role in food-seeking behaviour in rodents [41]. Our findings suggest that this mechanism is altered in individuals with increased BMI, as increased excitatory input from the insula to the hypothalamus was found irrespective of homeostatic state. This occurs together with reduced inhibition from the ventromedial prefrontal cortex to the anterior insula—a pathway that codes changes in incentive salience in response to changes in homeostatic state [42, 43]. In fitting with this interpretation, increased BMI would be associated with reduced neural sensitivity to changes in homeostatic/interoceptive state and related persistent attribution of salience to both hunger and satiety states. This argument is consistent with the eating without hunger framework [8], as the hypothalamus (regulating eating based in energy needs) is impacted by systems (i.e., ventromedial prefrontal cortex, anterior insula) encoding non-homeostatic aspects of food such as incentive salience and hedonic value.

Furthermore, we found that increased BMI was associated with changes in cortico-cortical interactions during the hunger state. Reductions in dorso-lateral prefrontal cortex influence over the ventromedial prefrontal cortex, as observed herein, play a central role in goal-directed food choice tasks and related dietary self-regulation [12, 44]. The interaction between dorso-lateral prefrontal and the ventromedial prefrontal cortex has been shown to play the key role in context-dependent valuation that requires self-control [45]. However, we cannot assume equivalence between the function of brain regions in task-related versus resting-state designs [46, 47], since these regions were activated by a food choice task in the same participants [11]. In the absence of more plausible alternative explanations, we speculate that these findings may relate to alterations in goal-oriented food choice. If our interpretation is correct, these results would align with an impaired self-regulation model of obesity [9, 10] but introduce the additional caveat that this mechanism may be state-specific as it was not observed in the satiety state. The increased BMI-related greater excitation from anterior insula and posterior cingulate regions over the dorso-lateral prefrontal cortex is consistent with this hunger-related effect [18].

Communication between ‘higher-order’ cortical regions and ‘lower-order’ subcortical areas (e.g., hypothalamus) is critical to governing feeding behaviour [48]. Our results in the hunger (versus satiety) state, irrespective of BMI, support a top-down interpretation of these relationships. This is in line with the notion that hunger triggers an incentive mechanism during energy depletion that motivates food seeking in order to avoid starvation [49, 50]. Studies in rodent and primate models similarly support the role of ventromedial prefrontal cortex-hypothalamus interactions in feeding behaviour [51]. Notably, we previously reported that homeostatic state influences local activity with the hypothalamus itself [11]. This observation is in line with the established sensitivity of the hypothalamus to the neuropeptides that signal and regulate energy needs (e.g., ghrelin and leptin; [52, 53]).

In conclusion, our study reveals changes in directed connectivity between prefrontal, insular, striatal and hypothalamic regions as a function of BMI and homeostatic state. Although the hedonic eating view has been a dominant account of obesity, our findings point to a model in which reduced sensitivity to homeostatic/interoceptive changes and disrupted prefrontal communication during caloric deprivation. Our results highlight the potential for intrinsic predispositions (‘neuromarkers’), to improve individualised intervention strategies that can either alter obesogenic traits or ameliorate detrimental effects such as weight gain. They also pave the way for developing non-invasive brain stimulation protocols aimed to rewire cortico-subcortical network abnormalities in obesity. Specifically, our findings provide proof-of-principle evidence to examine the impact of, for example, dorso-lateral prefrontal cortex stimulation and ventromedial prefrontal cortex inhibition on cortical-insular-hypothalamic network dynamics and obesity-related therapeutic outcomes. Our study is only the first step to deriving an understanding of this interplay of brain regions at rest; however, it cannot conclusively determine how these resting-state dynamics relate to these networks in action (e.g., feeding behaviour). At this stage, only one study has investigated the relationship between effectivity connectivity at rest and task and their relationship to behaviour [47]. Therefore, complementary future task-based studies will be required to examine how the revealed resting-state network dynamics translate during task performance.

## Supplementary information

Supplementary Material
