# Supplementary material for: Neural network modelling reveals changes in directional connectivity between cortical and hypothalamic regions with increased BMI
Source: Int J Obes (Lond). 2021 Aug 2;45(11):2447–54. doi: 10.1038/s41366-021-00918-y (PMC8528693; doi:10.1038/s41366-021-00918-y)
Supplement: Supplementary file 1 — Supplementary Material [file 41366_2021_918_MOESM1_ESM.docx]

**Supplementary Information**

**Supplementary Methods**

***Spectral Dynamic Causal Modelling***

Dynamic causal modelling (DCM) is Bayesian framework that infers the directed (causal) connectivity among the neuronal systems – referred to as effective connectivity. We recently proposed a new DCM for resting state fMRI – based upon a deterministic model that generates predicted cross spectra – referred to as spectral DCM. In order to model resting state activity – in the absence of external stimuli – we will have to add a stochastic component, i.e. neural fluctuations, to the classical DCM based on ordinary differential equations. Mathematically, we can express the formulation of the stochastic generative model using a set of two equations. First is the neuronal state equation, namely

$\dot{x}\left( t \right)=f\left( x(t),u(t),\theta\right)+ v(t)$, (S1)

and second is the observation equation, which is a static nonlinear mapping from the hidden physiological states in (1) to the observed BOLD activity and is written as:

$y\left( t \right)=h\left( x(t),\varphi\right)+ e\left( t \right),$ (S2)

where $\dot{x}(t)$ is the rate of change of the neuronal states $x\left( t \right)$, $\theta$ are unknown parameters (i.e., the effective connectivity) and $v(t)$ (resp. $e(t)$) is the stochastic process – called the state noise (resp. the measurement or observation noise) – modelling the random neuronal fluctuations that drive the resting state activity. In the observation equations, $\varphi$ are the unknown parameters of the (haemodynamic) observation function and $u(t)$ represents any exogenous (or experimental) inputs that drive the hidden states – that are usually absent in resting state designs ^1^. Spectral DCM furnishes a constrained inversion of the stochastic model by parameterising the neuronal fluctuations$v(t)$. Spectral DCM simplifies the generative model by replacing the original timeseries with their second-order statistics (i.e., cross spectra). This means, instead of estimating time varying hidden states, we are estimating their covariance which is time invariant. Then we simply need to estimate the covariance of the random fluctuations; where a scale free (power law) form for the state noise (resp. observation noise) is used – motivated from previous work on neuronal activity ^2,3^ – as follows:

$g_{v}\left( \omega,\theta\right)=\alpha_{v}\omega^{-\beta_{v}}$

$g_{e}\left( \omega,\theta\right)=\alpha_{e}\omega^{-\beta_{e}}$ (S3)

Here, $\left\{ \alpha,\beta\right\}\subset\theta$ are the parameters controlling the amplitudes and exponents of the spectral density of the neural fluctuations. The parameterisation of endogenous fluctuations means that the states are no longer probabilistic; hence the inversion scheme is significantly simpler, requiring estimation of only the parameters (and hyperparameters) of the model.

We used standard Bayesian model inversion to infer the parameters of the model in (1), (2) and (3), from the observed signal$y(t)$. The description of the Bayesian model inversion procedures based on variational Laplace can be found elsewhere for the interested readers ^4–6^.

***Parametric Empirical Bayes***

Empirical Bayes refers to the Bayesian inversion or fitting of hierarchical models. In hierarchical models, constraints on the posterior density over model parameters at any given level are provided by the level above. These constraints are called empirical priors because they are informed by empirical data. We recently introduced a second-level or between-subjects model over parameters, which represents how individual (within-subject) connections derive from the subjects’ group membership ^7^ – based on parametric empirical Bayes (PEB). This approach calls on Bayesian Model Reduction (BMR) to finesse the inversion of multiple models of a single dataset or a single (hierarchical) model of multiple datasets. BMR allows one to compute posterior densities over model parameters, under new prior densities, without explicitly inverting the model again. For example, one can invert a DCM for each subject in a group and then evaluate the posterior density over group effects, using the posterior densities over parameters from the single subject inversion. This may improve subject-specific parameter estimates, by using group-level estimates to rescue individual DCM from local optima. Mathematically, for DCM studies with *N* subjects and *M* parameters per DCM, we have a hierarchical model, where the responses of the *i*-th subject and the distribution of the parameters over subjects can be modeled as:

$y_{i}=\Gamma_{i}^{\left( 1 \right)}(\theta^{\left( 1 \right)})+ \varepsilon_{i}^{\left( 1 \right)}$ (S4)

$\theta^{\left( 1 \right)}=\Gamma^{\left( 2 \right)}\left( \theta^{\left( 2 \right)} \right)+ \varepsilon^{\left( 2 \right)}$

$\theta^{\left( 2 \right)}=\eta+ \varepsilon^{\left( 3 \right)}$

where, $y_{i}$is the BOLD time series from *i-th* subject *and* $\Gamma_{i}^{\left( 1 \right)}$ is a nonlinear mapping from the parameters of a model to the predicted response $y$ for e.g., as shown in Eq. S1 above. $\varepsilon_{i}^{(1)}$is independent and identically distributed (i.i.d.) observation noise (equivalent to $e\left( t \right)$ in Eq. S2). In this hierarchical form, *empirical priors* encoding second (between-subject) level effects place constraints on subject-specific parameters. The second level would be a linear model where the random effects are parameterised in terms of their precision:

$\Gamma^{\left( 2 \right)}\left( \theta^{\left( 2 \right)} \right)=(X\bigotimes W)\beta$

where, $\beta\subset\theta$ are group means or effects encoded by a design matrix with between $X$ and within-subject $W$parts. The between-subject part encodes differences among subjects or covariates such as age, while the within-subject part specifies mixtures of parameters that show random effects. We assume that the first column of the design matrix is a constant term, modelling group means, and subsequent columns encode group differences or covariates such as age.

**Supplementary References**

1 Friston KJ, Kahan J, Biswal B, Razi A. A DCM for resting state fMRI. *NeuroImage* 2014; **94**: 396–407.

2 Beggs JM, Plenz D. Neuronal Avalanches in Neocortical Circuits. *J Neurosci* 2003; **23**: 11167.

3 Stam CJ, de Bruin EA. Scale-free dynamics of global functional connectivity in the human brain. *Hum Brain Mapp* 2004; **22**: 97–109.

4 Friston K, Mattout J, Trujillo-Barreto N, Ashburner J, Penny W. Variational free energy and the Laplace approximation. *NeuroImage* 2007; **34**: 220–234.

5 A. Razi, K. J. Friston. The Connected Brain: Causality, models, and intrinsic dynamics. *IEEE Signal Process Mag* 2016; **33**: 14–35.

6 Friston KJ, Harrison L, Penny W. Dynamic causal modelling. *NeuroImage* 2003; **19**: 1273–1302.

7 Friston KJ, Litvak V, Oswal A, Razi A, Stephan KE, van Wijk BCM *et al.* Bayesian model reduction and empirical Bayes for group (DCM) studies. *NeuroImage* 2016; **128**: 413–431.
